# Pyrolysis of Fluorocarbon Polymers*

**DOI:** 10.6028/jres.065A.027

**Published:** 1961-06-01

**Authors:** Leo A. Wall, Sidney Straus

## Abstract

The thermal decomposition of various fluorocarbon polymers were investigated; volatile products of the decomposition were determined along with the overall rates of volatilization, and from these rates the activation energies were calculated for the thermal degradation reactions. The thermally most stable of all the polymers thus far studied are the completely fluorinated ones. However, evidence from a study of the decomposition of hexafluoropropylene telomers and from the study of a copolymer of tetrafluoroethylene and hexafluoropropylene suggests that the homopolymer of hexafluoropropylene, if it could be made, would be quite unstable. The photoinduced decomposition of polytrifluorochloroethylene was also investigated, and estimates of the activation energies were obtained for the various elementary steps of the decomposition mechanism. The photoinduced experiments indicated that mutual termination of the radical intermediates takes place and that a diffusion effect on depropagation becomes pronounced below 250 °C.

## 1. Introduction

Knowledge of the thermal decomposition mechanisms of fluorocarbon materials and the influence of molecular structure on their thermal stability is fairly limited. There is ample evidence that linear perfluoroaliphatic substances have a high thermal stability [1, 2] 1 but few if any results are available for branched and aromatic perfluorocompounds or for the various copolymers containing fluorine, which are becoming important commercially. The mechanism of depolymerization or decomposition of these materials, with the possible exception of polytetrafluoroethylene [3 to 7], is also in need of extensive investigation, since such information would be useful in understanding the problems involved in the development of thermally more stable materials. There are numerous possible polymeric structures not yet synthesized. Studies on small molecules reported and discussed in this article will aid, it is hoped, in establishing the polymeric structures that are desirable for high- temperature polymers and hence will narrow down the number of objectives for synthesis research.

## 2. Materials Studied

### Perfluoroamidine Polymers

These polymers were received from H. C. Brown of the University of Florida. They were prepared by heating perfluoroamidines [8] which then polymerized by splitting out ammonia and forming triazine rings. Three polymers were studied:

### Polymer A1

A polymer of perfluoroglutarodiamidine alone. It was a hard brittle solid and was not soluble in any of the usual organic solvents. It was presumably a highly cross linked material composed mainly of the following structure:

**Figure:**
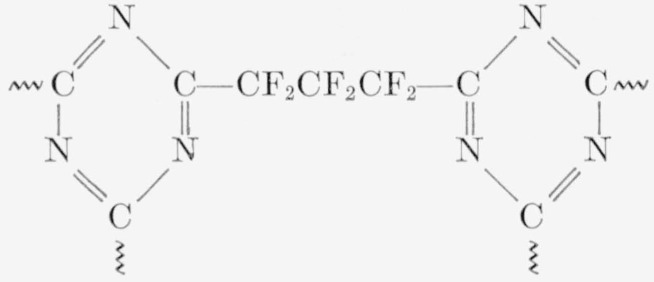


### Polymer A2

A copolymer of perfluoroglutarodiamidine and perfluorobutyroamidine in a 1 to 1 molar ratio. It was a milky white, somewhat elastomeric solid, slowly soluble in pyridine and in ethylene diamine, with mainly the following structure:

**Figure:**
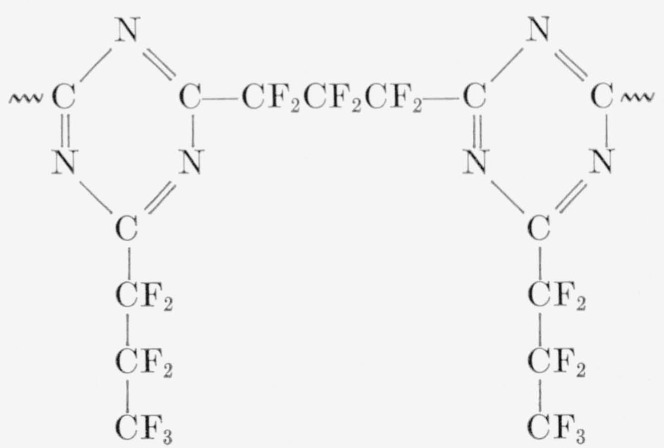


The polymer must, of course, be highly branched. A few solution measurements tended to support this conclusion. Viscosity measurements were made using pyridine as a solvent. Solution of the sample required 5 to 6 days at room temperature using a wrist-action mechanical shaker. The concentration of the solution was 1.53 wt-percent. Viscosity measurements were made on the 6th, 13th, 20th, and 27th day using a Ubbelohde viscometer. On the 6th day the specific viscosity was 0.094. On the 13th day the specific viscosity had dropped to 0.044 at which value it remained even after 27 days. Assuming the molecules are spherical, one calculates a specific viscosity near 0.03. In view of the method of synthesis for this polymer, the close agreement between the calculated value and the experimental measurements suggests a highly branched molecule. The disaggregation indicated by the viscosity measurements between the 6th and 13th day may mean that solution in pyridine occurs with slow degradation. The material when recovered from solution was a brown wax and had none of the rubbery character of the original material.

### FEP 100–X

A copolymer of tetrafluoroethylene and hexafluoropropylene. It is a thermoplastic material available from the DuPont Co.

### VFHP–A

A copolymer of vinylidene flouride and hexafluoropropylene. It is a milky-white elastomeric gum available from the DuPont Co.

### F

A polymer of chlorotrifluoroethylene. It is a thermoplastic material available from Minnesota Mining and Manufacturing Co. The sample used was Grade 300 molding powder with a reported number-average molecular weight of approximately 350,000. Data from previous work [10] on other samples of this polymer are used for comparison purposes in this article.

### F 3700

A copolymer of chlorotrifluoroethylene and vinylidene fluoride. It is a white elastomeric gum. From analysis which showed that it contained 11.3 wt percent chlorine, it was calculated to be 24.4 mole percent chlorotrifluoroethylene.

### F 5500

A copolymer similar to F 3700. Analysis showed that it contained 15.5 wt percent chlorine or 36.2 mole percent chlorotrifluoroethylene.

### Polymer A3

A copolymer of perfluoroadipodiamidine and perfluorobutyroamidine in a ratio of 1 to 1.35. This copolymer was also a milky white, somewhat elastomeric substance, slowly soluble in pyridine. Its structure was presumably the same as that for A2, except for the additional difluoromethylene group, CF_2_, in the diamidine unit. The compounds A2 and A3, when first prepared, were yellow tacky gums which became milky white some what elastomeric substances when heated in the air for several days at 350 °C. This behavior was somewhat erratic for certain samples of the yellow gums which when stored for several months failed to cure to white elastomeric substances when heated to 350 °C. Also heating for longer times tended to remove entirely the elastomeric character. The data reported here are for the two copolymers after curing at 350° C. The thermal stability of uncured samples was much poorer, indicating that curing does not occur under the vacuum conditions used in the test, at least not without a large amount of the material volatizing.

### Polyhexafluoropentylene adipate

An experimental polymer [9] made at the Hooker Electrochemical Co. having the formula

**Figure:**
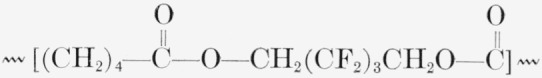


It is a milky-white tough gum with a reported number-average molecular weight of approximately 17,000.

### Polytrifluoroethylene

Prepared for this study by polymerizing trifluoroethylene in a rocking bomb using aqueous persulfate as a catalyst and heating for 24 hr at 160 °C.

### Telomers of Hexafluoropropylene

The pyrolysis of the following telomers of hexafluoropropylene [11,12] was also investigated.

### Telomer PPF9A

A white wax having a number-average molecular weight of 1,541. Its average structure is represented by the formula

**Figure:**
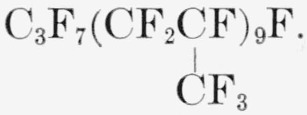


### Telomer PPF6.5A

A clear viscous grease with the average composition 
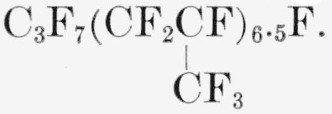


### Telomer PPC16A

A clear viscous grease with the average composition of 
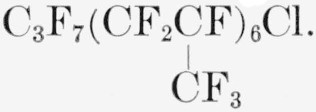


### Telomer PPC2A

A clear liquid with the average composition 
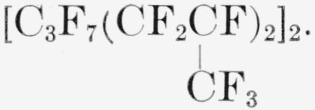


### Telomer BVFC5A

A clear liquid with the average composition 
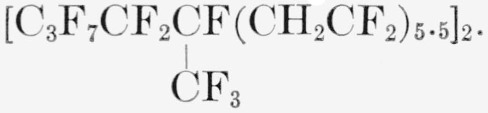


## 3. Experimental Procedures

For the investigation of the thermal decomposition of the high polymers, two types of measurements were made. The first involved the identification of the more volatile products of decomposition by mass spectrometry. The apparatus and technique employed have been fully described previously (13). A 15–40 mg sample was pyrolyzed in an evacuated glass tube inserted into a furnace maintained at constant temperature. The sample was heated for 30 min. The weights of four fractions were obtained:

1. A “residue”, i.e., that which remained in the furnace;
2. a “waxlike fraction”, volatile at the temperature of pyrolysis;
3. a “condensed fraction”, volatile at room temperature;
4. a “gas fraction”, volatile at liquid nitrogen temperature.

Fractions 3 and 4 were then analyzed by mass spectrometry when adequate calibration patterns were available. The distribution of pyrolysis products into these four fractions gave an insight into the mechanism because it establishes whether polymers tend to give large fragments or small ones, which are often the monomers. In addition, the 30-min weight losses obtained by this technique provided a useful method of comparing polymers for thermal stability, and it also established the temperature range to be used for the second type of measurement, the rate study.

The apparatus and experimental procedure for obtaining the rates of thermal decomposition for high polymers have been described in detail [14, 15]. It is essentially a technique for measuring the weight loss as a function of time. A very sensitive tungsten helical spring balance is used to suspend a 5 to 6 mg sample in an evacuated space. A small platinum or glass crucible is used to contain the polymer. The glass crucible gives better results when the sample does not melt and flow, presumably because radiant heat passes through the glass. The deflection of the tungsten spring is determined by means of a cathetometer. A thermoregulated furnace is raised to surround the suspended sample at the beginning of an experiment. A graph is made of the weight loss as a function of time, from slopes of which the rates at various times or conversions are obtained and plotted. From the shapes of these characteristic curves certain aspects of the thermal decomposition mechanism are obtainable [16–18].

The procedures described above were not applicable to the low molecular weight telomers of hexafluoropropylene. These materials would merely distill without decomposing. A variation of a technique that is often used [19, 20] for small molecules such as in a recent investigation of the thermal decomposition of certain phosphinoborines [21], was therefore employed.

The procedure used for the hexafluoropropylene telomers was as follows:

Samples of 100 ±1 mg were weighed in a small bucket, and the bucket placed inside a 10 ml ampule. Ampules and weighing buckets of both glass and nickel were used in order to ascertain whether the container composition influenced the thermal decomposition. These ampules were then evacuated, generally eight at a time, to 10^−4^ mm of mercury. After evacuation, argon gas was introduced simultaneously into all eight tubes. The gas was previously purified by passing through a silica gel trap cooled to 197 °K in order to eliminate, as much as possible, water and oxygen. The amount of argon introduced was sufficient to give a pressure of about 50 to 100 mm of mercury. The moles of argon in each tube thus corresponded to approximately three times the moles of material to be pyrolyzed, and each tube contained to within ± 1 percent the same amount of telomer and argon. After introduction of the argon, each tube was sealed off. The nickel ampules were connected via a Kovar- Pyrex seal to a Pyrex tube. The ampules were pyrolyzed in a large copper furnace thermoregulated to ± 1 °C. The pyrolysis products and also a sample of the argon were analyzed by means of a mass spectrometer, and the ratio of product to argon permitted the calculation of yield of the products of pyrolysis.

## 4. Results

Pyrolysis of the three perfluoroamidine polymers for 30 min periods gave the data shown in table 1. It was found that quite high temperatures were required to cause an appreciable amount of volatilization. The first two columns list the temperature of pyrolysis and the initial weight of sample. The third column gives the weight loss in percent of original sample, and the final column gives the percent of the total products that were volatile at room temperature. The gas fraction was negligible and is not listed. The percent of heavy wax can be obtained by subtracting the percent light volatiles from 100. The residues in all cases were brown to black spongy solids, depending on temperature. The wax fraction which was brown in color, was obtained in greatest quantity from the elastomeric A3 material and least from the most cross linked brittle A1.

Mass spectrometric analysis of the light volatiles from the perfluoroamidine polymers are shown in table 2. The data for the A2 polymer were obtained at 489 °C, those for the other two at 470 °C. For all three polymers the major product found in the light volatiles was tetrafluoroethylene, the actual yields of which in weight percent of the total amount volatilized were 50, 20, and 4, respectively, for the polymers A1, A2, and A3. It is somewhat surprising that no nitrogen-containing product was identified in the light volatile fraction. The silicon tetrafluoride must come from reaction with the glass in the pyrolysis apparatus, either via the formation of hydrogen fluoride or from the reaction of the fluorocarbon fragments with the glass at the high temperatures of the furnace. The latter reaction may also account for some of the CO_2_ found with the A3 polymer. The extent of reaction with the glass would be expected to depend, among other things, on the temperature. At 500 °C the A2 polymer showed 12 mole-percent silicon tetrafluoride in the light volatiles. In addition to the listed compounds, about 10 wt-percent of the light volatiles were unidentifiable compounds. The hydrocarbons listed for A1 must be the result of some solvent contamination. Most of the carbon dioxide probably comes from carboxyl groups in the polymer derived from the original amidine groups.

Infrared spectra were obtained on the original polymers, A1 and A2, and on their residues at about 11 to 14 wt-percent pyrolysis. The spectra in each case for the original and pyrolyzed polymer were essentially identical with the exception of minor differences related to particle size and clarity of the KBr pellet used in the infrared technique. There was, however, a small carbonyl band at about 5.7 *μ* in the original samples which was eliminated during the pyrolysis.

Without analyzing or collecting products, a few longtime weight losses were measured on the A2 polymer at 355 °C and 400 °C. At 355 °C the polymer was heated for 180 hr, 136 hr of which were in a vacuum and the remainder in air. The sample lost 2.6 percent of its weight in the first hour and 5.2 percent during the remaining 179 hr. At 400 °C the polymer upon heating 41 hr in a vacuum lost 3.1 percent in the first hour and 7.7 percent of its initial weight in the remaining 40 hr. Thus, neglecting the weight losses in the first hour, the rate of weight loss at 355 °C and 400 °C were, respectively, 0.00048 and 0.0032 percent/min. These results agree quite well with those calculated using the Arrhenius relationship and the data obtained from the rate studies in the region of 500 °C. At 355 °C the calculated rate is 0.00054 percent/min.

**Figure 1:**
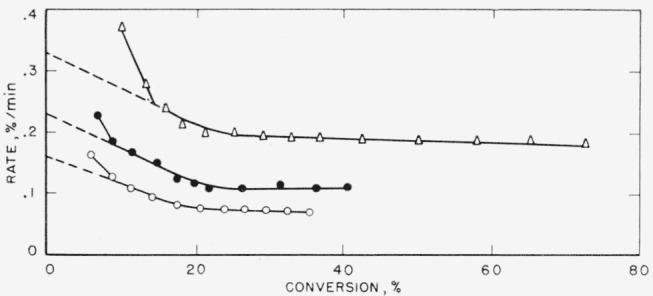
Rate of volatilization versus conversion for polymer A1 Δ, 513 °C; ● 501 °C; ○, 491 °C.

**Figure 2:**
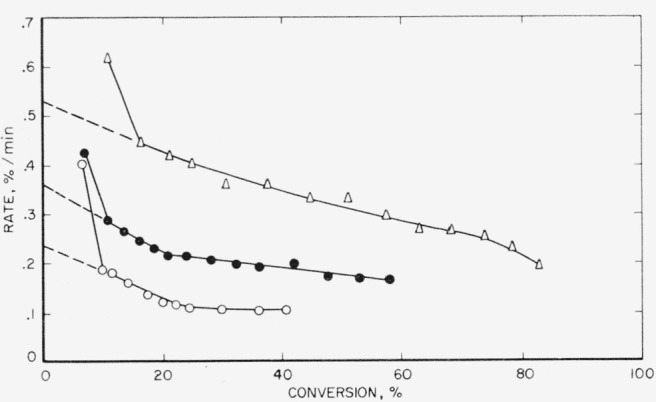
Rate of volatilization versus conversion for polymer A2 Δ, 513 °C; ● 502 °C; ○, 491 °C.

**Figure 3:**
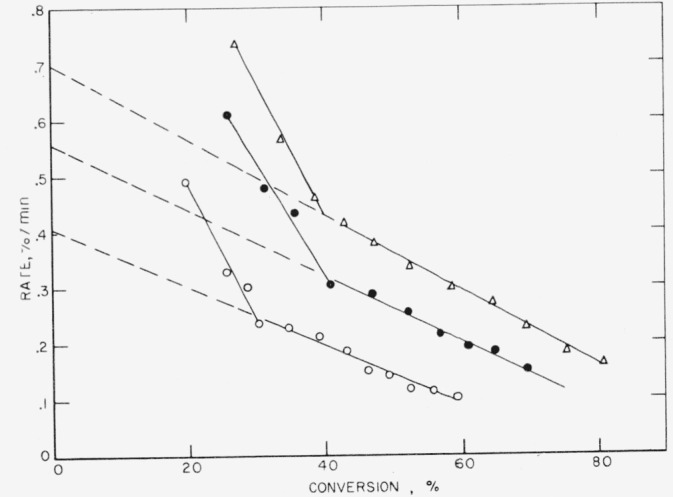
Rate of volatilization versus conversion for polymer A3 Δ, 500 °C; ● 490 °C; ○, 480 °C.

In figures 1, 2, and 3, the rates of volatilization of the perfluoroamidine polymers are shown as a function of the conversion to volatile products. The shapes of these rate curves indicate a rather complex mechanism. Initially very fast, they drop at 20 to 30 percent conversion to a value which tends to be constant up to about 70 percent conversion. Arbitrary extrapolations were made as shown by the dashed lines to obtain an apparent initial rate. From such initial rates activation energies were calculated. Straight-line Arrhenius plots were obtained. Extrapolation from higher conversion portions of the curves (see figs. 1 and 2) would not greatly alter the activation energy calculated.

A study was made of the effect of small amounts of acid and methanol on the pyrolysis of the A2 polymer. After various treatments, the polymer was dried, preheated to 110 °C in vacuum for 1 hr, and then pyrolyzed at 500 °C. A small sample held in methanol containing 0.2 percent sulfuric acid for 50 days doubled the rate. However, methanol alone for 50 days also doubled the rate and a methanol soaking for 7 days increased the rate by 50 percent. Methanol treatment for 30 min had no effect on the rate, i.e., the initial rate determined by extrapolation (see fig. 2). Methanol treatment tended to slow the actual high initial rate. The polymer A2 could be swollen in methanol, though it does not dissolve. Possibly small molecules were washed out by the methanol treatments. Polymer swollen by methanol crumbled easily on touching, but when redried shrank back to its original shape and had apparently all its original elastomeric properties. All samples immersed in methanol slowly became yellow in color. The longer the material was immersed, the more intense was the color. The sample treated with methanol for 30 min became black when heated in vacuum at 400 °C for 1 hr. After a similar heating, an untreated sample would be tan in color.

These results suggest that methanol has a definite degrading effect on the polymer. Water in the methanol may be partly involved in the degradation, except that water alone would not swell the material.

### 4.1. Polyhexafluoropentyleneadipate

**Figure 4:**
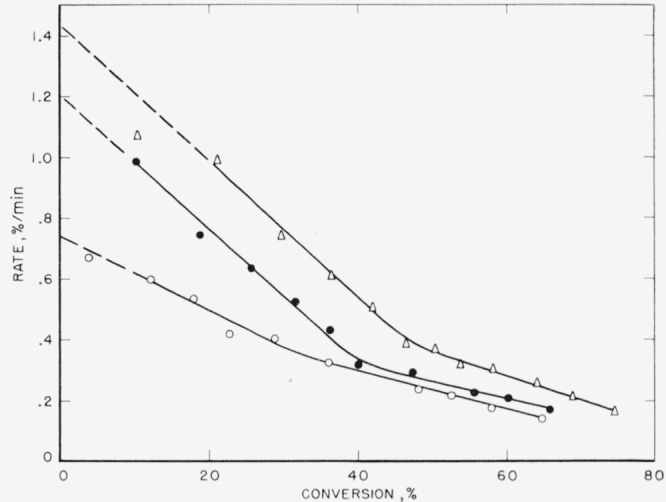
Rate of volatilization versus conversion for polyhexafluoropentyleneadipate Δ, 345 °C; ●, 340 °C; *○*, 330 °C.

Pyrolysis of this material produced only a small quantity of light volatiles (see table 3). About 30 percent of the light volatiles for this polymer could not be identified; some of them contained fluorine, and the remainder were hydrocarbons and carbon dioxide. The relative proportions of the identified compounds are given in table 4. One would have expected some silicon tetrafluoride from this polymer. The rate of volatilization as a function of conversion is presented on figure 4. This polymer shows an unexpected rate behavior. Polyamides and polyesters usually produce rates that pass through a maximum [22, 23] when plotted against conversion or time.

### 4.2. FEP 100–X

This material produces somewhat less light volatiles (see table 3) than pure polytetrafluoroethylene, TFE, 75 to 84 percent as compared to about 95 percent for the latter. The main products (see table 4) are the two monomers tetrafluoroethylene and hexafluoropropylene, the latter being more abundant in the initial stages of degradation. At 10 percent decomposition the light volatiles analyzed 85 percent C_3_F_6_ and 9 percent C_2_F_4_, while at 92 percent decomposition analysis gave 19 percent C_3_F_6_ and 68 percent C_2_F_4_. From the results at the higher conversion it seems evident that the composition of FEP 100–X is about 22 mole percent hexafluoropropylene and 78 mole percent tetrafluoroethylene.

**Figure 5:**
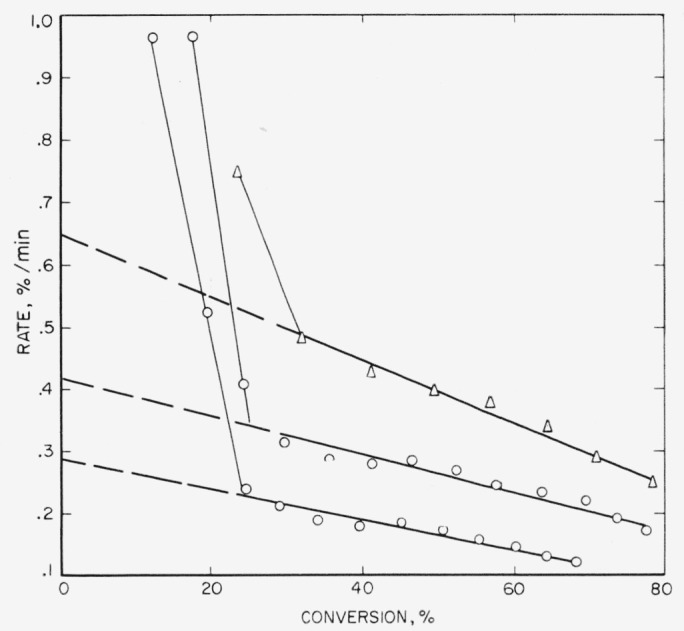
Rate of volatilization versus conversion for FEP 100 X Δ, 508 °C; ●, 499 °C; ○, 490 °C.

As seen also from the rate behavior (fig. 5), there appear to be two stages in the degradation. The rate at first drops quickly, but then after about 30 percent conversion it decreases much more slowly. With the exception of the first portion, the rate curves are similar to those found for polytetrafluoroethylene [3] and are compatible with the relatively high yield of monomer [16 to 18]. The initial more rapid degradation suggests a much lower stability of chain segments containing the hexafluoropropylene units. Also the somewhat lower monomer yield suggests more of the transfer type reactions [18] than occur in the pyrolysis of polytetrafluoroethylene.

### 4.3. VFHP, F 3700, and F 5500

**Figure 6:**
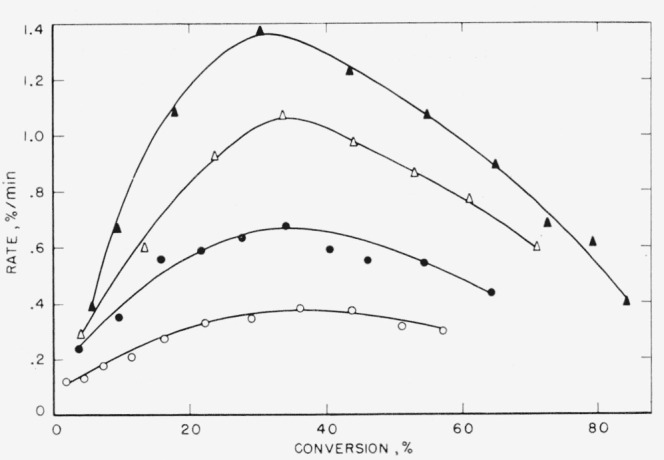
Rate of volatilization versus conversion for VFHP–A ▲, 405 °C; Δ, 400 °C; ● 395 °C; ○, 385 °C.

**Figure 7:**
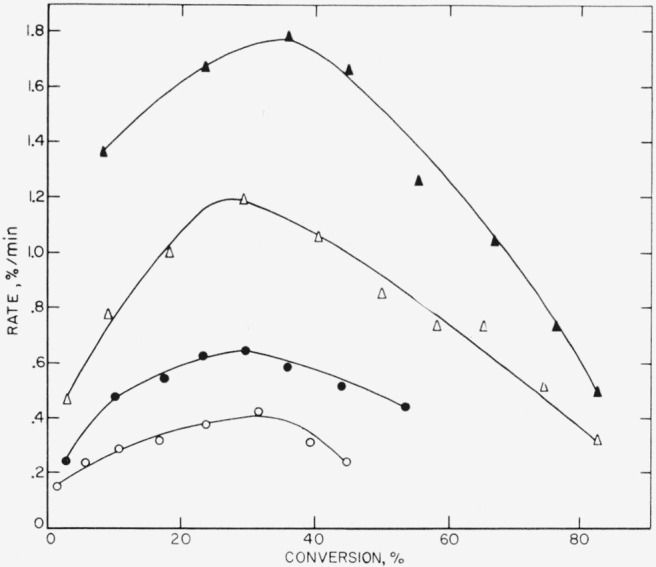
Rate of volatilization versus conversion for F 3700 ▲, 395 °C; Δ, 389 °C; ●, 380 °C; ○, 375 °C.

**Figure 8:**
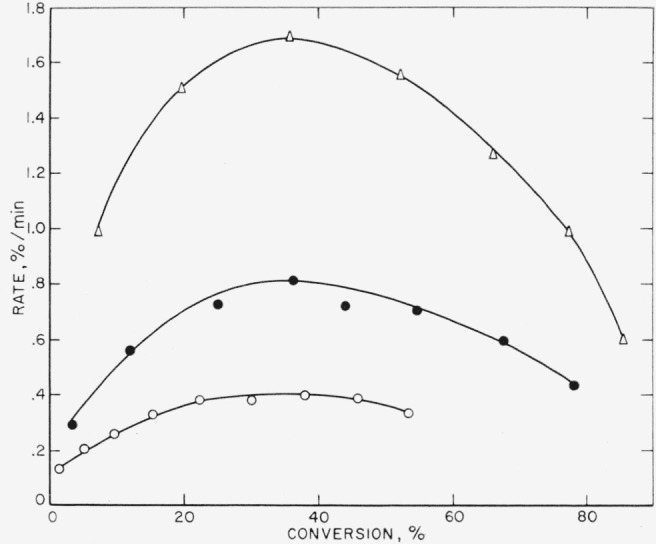
Rate of volatilization versus conversion for F 5500 Δ, 395 °C; ●, 385 °C; ○, 375 °C.

These three polymers gave very similar results on thermal decomposition, which is not surprising since all three are copolymers containing vinylidene fluoride as one of the monomers. In table 3 it is seen that the amount of light volatiles decreases in the order in which the polymers are listed above, or conversely, the amount of heavy volatiles or wax- fraction increases. The major volatile product is hydrogen fluoride, which reacts with the glass of the apparatus to form the silicon tetrafluoride listed in table 4. The rate curves (figs. 6,7, and 8) are similar in character, all showing maximums which suggest that the mechanism of breakdown can be considered to be approximately a random one. An ideal random degradation would have the maximum occurring at 26 percent conversion. About the same range of temperatures is used to study the decomposition of all three polymers.

### 4.4. Polytrifluoroethylene and Poly-1,1-difluoroethylene

**Figure 9:**
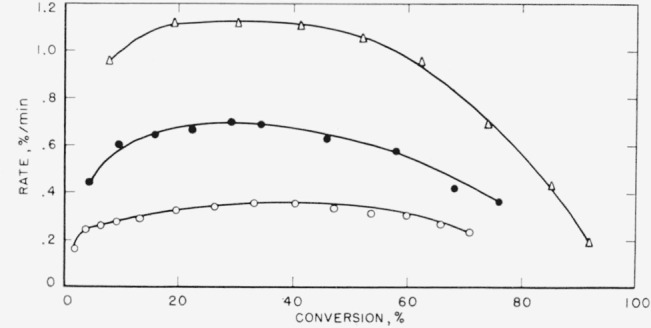
Rate of volatilization versus conversion for polytrifluoroethylene Δ,420 °C; ●, 410 °C; ○, 400 °C.

Polytrifluoroethylene has been partially investigated [24] previous to this work. Its major products of thermal decomposition are hydrogen fluoride and heavy unidentified species found in the wax fraction which comprised about 80 percent of the material volatilized during decomposition. The rate data are presented in figure 9. The curves are quite close in shape to those predicted for an ideal random decomposition of the main chain. Another process dehydrohalogenation, is, of course, simultaneously occurring, as is evidenced by the production of appreciable hydrogen fluoride [24].

Poly-1,1-difluoroethylene has been studied by other workers [25]. It produces more copious quantities of hydrogen fluoride and leaves a charredappearing residue amounting to about 40 percent of the polymer sample pyrolyzed. However, over the first 60 percent of conversion the rate curves resemble those for polytrifluoroethylene in that a pronounced maximum in the rate occurs. Neither of these two polymers produce more than trace amounts of monomer.

### 4.5. Polytrifluorochloroethylene

**Figure 10:**
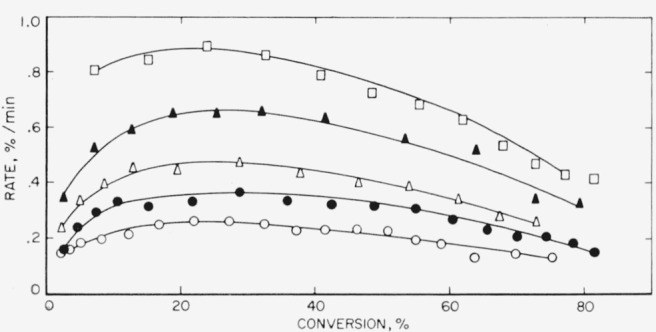
Rate of volatilization versus conversion for polytrifluorochloroethylene □, 371 °C; ▲, 366 °C; Δ, 361 °C; ●, 356 °C; ○, 351 °C.

Polytrifluorochloroethylene (F) has been the subject of a previous study [10]. The major fraction, 70 wt percent, produced was the wax or heavy fraction. The light volatiles amounted to approximately 28 wt percent of the material volatilized and was mainly monomer. The temperatures of previously reported rate studies have recently been corrected [26] and hence the activation energy also was corrected. The same data have been replotted in the manner used in this article and are presented in figure 10. The curves are again typical for random degradation and exhibit maximums in the rates. Our evaluation of the activation energy, discussed later in this article, is based on the maximum rates and is somewhat lower than all other values reported [10, 18, 26].

**Figure 11:**
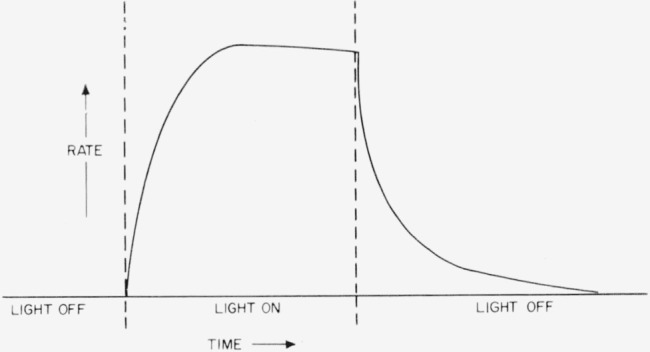
Rate behavior during UV irradiation and post irradiation.

**Figure 12:**
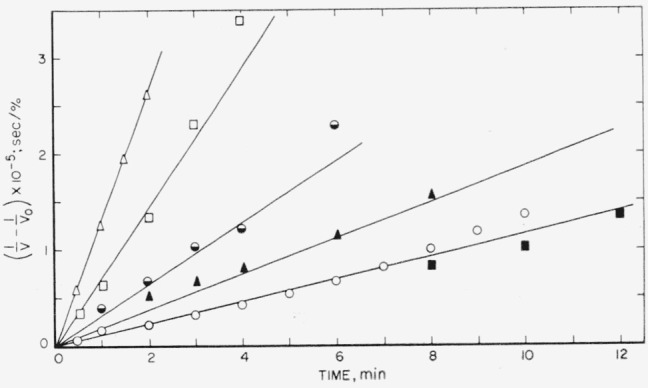
Decomposition of polytrifluorochloroethylene during the post-irradiation period (V=rate of monomer production) ■, 294 °C; ○, 277 °C; ▲, 247 °C and 261 °C; ○, 242 °C; □, 225 °C; Δ, 217 °C.

The photoinduced thermal depolymerization of F was explored using a medium-pressure quartz mercury lamp and a quartz window on the pyrolysis chamber. The apparatus was similar to that used previously [27], and a recording Pirani gauge was used to measure the rate of evolution of the light volatiles. The rate as recorded followed the pattern schematically shown in figure 11. Under appropriate conditions of temperature, usually about 100 °C below the normal pyrolysis temperatures, all polymers so far studied behave to some extent in the manner shown. The polymer, F, gave a fairly strong response to the ultraviolet light used and was studied in some detail because its light volatile fraction is mainly monomer. When the polymer is exposed to the light the rate rapidly rises to an essentially constant rate, which drops relatively slowly when the light is turned off. The time of the light and dark periods was roughly about 30 min. From both the photo rate, during continuous exposure to the light, and from the rate during the post-irradiation period, valuable information is obtained about the mechanism of decomposition. During post-irradiation the rate is in theory proportional to the concentration of intermediate radicals. From the time dependence of this rate, one can establish how this radical terminates. If the radical terminates bimolecularly, then a plot of the reciprocals of the rate of monomer production, V, during the post-irradiation against time should yield straight lines. In figure 12, such plots are shown for a series of temperatures. All things considered, the fit of the curves is excellent. Over the same time range a first-order plot of the same data gave markedly curved lines.

Polytetrafluorethylene showed only a feeble though definite response to the ultraviolet light used. This polymer does not absorb light except at shorter wavelengths and hence the result was not unexpected. The photo rate observed was probably the result of light absorption via impurities since, during successive runs on the same sample, the photo rate markedly decreased. Further experiments on this polymer are planned with shorter wavelengths and more intense ultraviolet radiation.

### 4.6. Telomers of Hexafluoropropylene

The telomer designated as PPF9A was studied the most extensively since it had the largest molecular weight among the telomers listed earlier. Decompositions were carried out in sealed tubes containing the materials with argon as an internal reference, so that mass spectral analysis would provide data on the extent of decomposition. At present, data on the mass spectra of fluorocarbons are fairly limited. Spectra of only twelve of the possible products were available for interpreting and computing the results. The products on the whole comprised a fairly complex mixture, and a complete analysis of the products could not be obtained. They were, however, paraffinic and olefinic fluorocarbons containing 1 to 6 carbon atoms per molecule. The original material contained an average of 30 carbon atoms, so these results indicate that decomposition takes place to give relatively small molecules.

Initial experiments were carried out at 350 °C; extensive decomposition occurred in the first 2 hrs and no marked change occurred at longer times. Other experiments were conducted at lower and at higher temperatures. The mass spectra showed that the main component in the products was hexafluoropropylene. From the amount of argon in the tubes and the weight of sample, it was estimated for the 350 °C run that 3 moles of hexafluoropropylene were produced per mole of starting material, i.e., about a 30 percent yield of this compound in 2 hr. Longer times gave the same result at this temperature.

The trend of decomposition at various temperatures can be seen from the data in table 5, where the ratio of the CF_3_^+^ ion to argon found in the mass spectra of each pyrolysis experiment is listed. Experiments were also performed at 150 °C, but no decomposition occurred. From table 5 it is seen that decomposition occurs as low as 200 °C, becomes appreciable at 250 °C for 24 hr, and appears to be essentially complete at 350 °C in less than 2 hr. The nickel tubes seem to give a little cleaner reaction and show slightly greater yields of hexafluoropropylene. However this effect is not large enough to change the general conclusions based on work with glass containers. It appears that at 350 °C and above the hexafluoropropylene reacts appreciably with the glass. At 8 and 24 hr at 350 °C and in all runs at 400 °C small amounts of silicon tetrafluoride were produced.

The results on the pyrolyses at 225 °C of the other telomers are shown in table 6. Their thermal stabilities appear to differ little from the PPF9A material, the latter being perhaps the least stable by a small degree. For the material PPC16A, which contains one chlorine atom per molecule of telomer, the mass spectra showed a small quantity of HCl^+^ ions. The telomer BVFC5A, which contains some hydrogen, produced considerable hydrogen fluoride and little or no hexafluoropropylene.

## 5. Discussion

The fluoropolymers examined in this investigation decompose by a wide variety of mechanisms. Before discussing the polymers studied, which in the main are copolymers, it will be useful to compare the thermal decomposition of fluorine-containing homopolymers for which data are available. For convenient comparison, the essential facts concerning the degradation of known fluorohomopolymers are listed in table 7. The replacement of fluorine in the Teflon structure by a phenyl group, chlorine atoms, or hydrogen atom is seen to change greatly the mechanism of decomposition and the thermal stability. The phenyl group produces the least change in monomer yield but shows the greatest change in thermal stability, although the activation energy is not greatly decreased. The monomer yield and the rate behavior give some insight into the mechanism of decomposition [16 to 18]. The mechanism for Teflon and poly-*α*, *β*, *β*-trifluorostyrene involves relatively long zip lengths [17], as indicated by the monomer yield and rate of volatilization curves [18]. Elucidation of other details of the mechanism such as the mode of initiation requires additional experiments not yet available for any fluoropolymers [28].

From the variation of rate with molecular size one can sometimes deduce whether the initial cleavage is at the end or at random along the chain. For polytetrafluoroethylene, studies of this type have so far been inconclusive, the rate being independent of molecular size [4, 6, 28]. Numerous polymers of tetrafluoroethylene prepared in various ways with various catalysts, including fluorine gas, all have essentially the same rate of volatilization [4].

Activation energies and estimated values of the preexponential factors are presented in tables 7 and 8, as well as the rates of volatilization at 350° C. For the purpose of calculation of the preexponential constants, rate data on all polymers not exhibiting a maximum in the rate are extrapolated to zero conversion in some manner which usually eliminates initial effects. The extrapolated values are then assumed to be the overall rate constants for thermal decomposition and assumed to be first order. For details and various possible relationships between the intercept and mechanisms reference should be made to various theoretical treatments [11, 17]. With random initiation the rate constants, and hence the preexponential factors, may contain a factor, *N*, the degree of polymerization of the decomposing molecules. Thus the extremely high values for the preexponential factors, 10^19^ listed in table 7 may indicate random initiation. However, correcting them by even the highest possible factor, 10^5^, the 10^14^ values so obtained are still among the highest. With Teflon, low-molecular weight polymers gave the same rate values as those of high molecular weight; thus deductions based on this apparently high preexponential factor are extremely tenuous.

The substitution of a chlorine atom in the tetrafluoroethylene unit completely changes the decomposition in all respects. The rate of volatilization curve suggests a net random decomposition mechanism. Very likely a chlorine atom dissociates off the chain in the initiation step, followed by chain rupture and some depropagation. Competing with depropagation are transfer reactions in which a depropagating radical abstracts a chlorine atom from another chain. Some transfer, of course, may be intramolecular. The net effect, as seen from the rate of volatilization curve (fig. 10) and the products of decomposition, is a net random degradation. In such cases the maximum in the rate curve is used to calculate the pre-exponential factor and activation energy. Also, the rates at 350 °C listed in tables 7 and 8 are the maximum rates. All polymers exhibiting the maximum-type curve are treated according to the theory for random decomposition [16, 29].

The rate at the maximum, *(dc/dt*)_max_, is related by the expression, *dc*/*dt*=*kL*/2.7, to the apparent first-order constant, *k*, for random cleavage of the bonds of the polymer chains. This random cleavage may be by simple unimolecular dissociation or the net effect of a chain reaction where intermolecular transfer is the chain-carrying process. The parameter, *L*, is the critical size for decomposition. In the type of open system in which these thermal decompositions are carried out, polymers with fewer than *L* units evaporate without decomposition [29]. Normally it is assumed that hydrocarbon molecules of molecular weight greater than 1,000 and fluorocarbon molecules greater than 2,000 cannot distill without decomposition. For the polymers with maximum-type curves (figs. 6, 7, 8, 9, and 10) it is from *k*, calculated by the above expression, that the values of *A* and *E* shown in tables 7 and 8 are obtained.

The materials for which data are listed in table 8 are in the main copolymers, although strictly speaking the amidine A1 and polyhexafluoropentyleneadipate should not be considered as such. The latter polymer and all the amidine polymers show both rather low activation energies and low pre-exponential factors. This, in conjunction with the effect of methanol in causing degradation, suggests a mechanism of thermal degradation for the amidine polymers at least partly hydrolytic. These polymers must contain many free amidine groups which become hydrolyzed in the presence of the moisture normally present in the atmosphere. At pyrolytic temperatures, entrapped or bound moisture may play an important role in the degradation. The low *A* and *E* values for polyhexafluoropentylene adipate are reminiscent of earlier studies of polyamides and polyesters [22, 23], where hydrolysis is a factor in thermal decomposition. The presence of fluorine in a polyester is quite likely to increase the ease of hydrolysis. Even if no other effect were responsible for the ease of presumably hydrolytic decomposition of polyhexafluoropentylene adipate, its structure can lead to hydrofluoric acid formation which would catalyze hydrolysis. Very small amounts of water contamination would then be responsible for the observed results.

FEP 100–X shows a much lower activation energy than polytetrafluorothylene. The stability of a polypropylene chain, as seen from the results of the pyrolysis of the telomers, is apparently rather low. Thus, the incorporation of hexafluoropropylene in the chain leads to considerable loss in stability, as indicated by the lower activation energy (compare polytetrafluoroethylene, table 7, with FEP 100–X, table 8). However, the pre-exponential factor is also a great deal lower, and hence thermal rates are not greatly different.

In the copolymer 100–X, the isolation of hexafluoropropylene units between tetrafluoroethylene units may, of course, minimize the deleterious effect of the hexafluoropropylene. The copolymers of 1,1-difhiorothylene,VFHP-A, F3700, and F5500, have roughly the same rate of decomposition at 350 °C as poly-1,1-difluoroethylene. Although the activation energy for the decomposition of polyhexafluoropropylene is unknown, it is likely to be considerably less than the 55 kcal value for FEP 100–X. Assuming this, it is interesting to note that the activation energies of all the 1,1-difluoroethylene containing copolymers are greater than those of the homopolymers of either of their monomers. At lower temperatures the copolymers would be slower to decompose, and there appears to be then a small mutual inhibition effect for thermal decomposition between the different monomer units.

**Figure 13:**
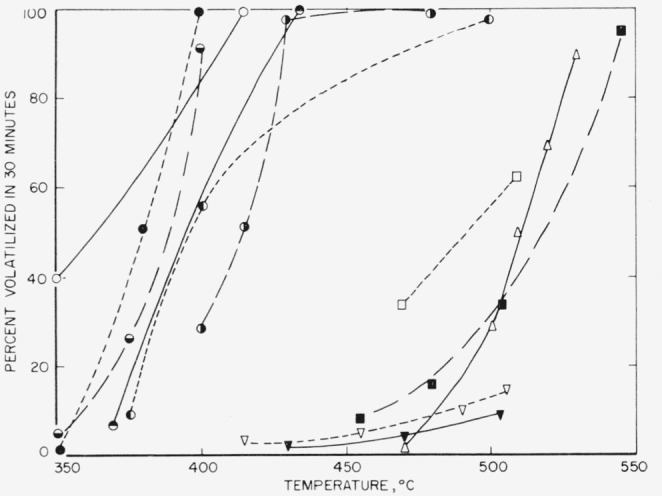
Thermal decomposition of fluoropolymers; amount volatilized in thirty minutes at various temperatures ○, Polyhexafluoroethyleneadipate ●, F *◒*, F 3700 *◓*, F 5500 *◐*, VFHP-A ◑, Polytrifluoroethylene □, Amidine, A3 ■, FEP 100 X ▽, Amidine A2 ▼, Amidine A1 △, TFE6 [3]

**Figure 14:**
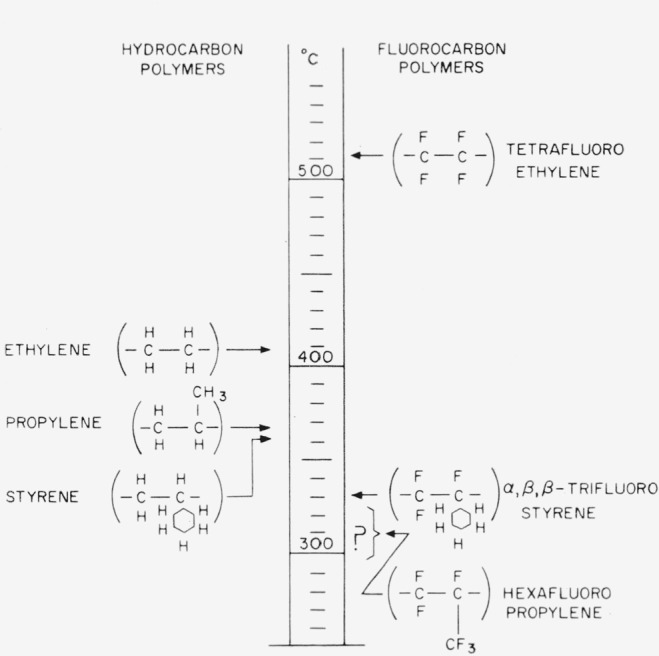
Comparison of the thermal stabilities of hydrocarbon with fluorocarbon polymers temperature at which rate of volatilization is 1 percent/minute.

For a practical comparison of the relative thermal stability of the polymers studied here see figure 13, in which the weight loss for 30-min periods is plotted against temperature. It is immediately evident that the polymers divide according to thermal stability into two groups. The most stable group consists of those which are perfluorinated. The least stable group consists of those polymers containing some hydrogen or chlorine atoms. The best of this group is no better than polyethylene. However, though it seems clear that incompletely fluorinated polymers are unlikely to be exceptionally stable, i.e., no better than the better hydrocarbon materials, the results with the hexafluoropropylene telomers suggest that some polymers containing branched perfluorinated structures are likely to be extremely unstable. Hence, perfluorination per se will not necessarily lead to a more stable structure than its hydrocarbon analogue. From the sparse amount of data available, it appears that structural variations in the fluorocarbons lead to a greater span of stabilities than similar variations in the hydrocarbon. In figure 14 the temperatures at which the listed polymers decompose at a rate of volatilization of 1 percent/min are indicated. Several hydrocarbon polymers are compared to several fluorocarbon polymers. The temperature indicated for polyhexafluoropropylene is an educated guess based on the results with the low molecular-weight telomers.

Unfortunately, thermal decomposition studies on fluorocarbon polymers are limited by the lack of polymers. Since the synthesis of a truly high polymer is often more difficult than smaller molecules, some studies have been made on smaller molecules to ascertain the appropriate approach to a high- temperature polymer. Early work on small fluorocarbon molecule studies is mainly that of Cady [1]. He carried out gas-phase pyrolysis of hexafluoroethane, decafluorocyclopentane, octafluoropropane, decafluoro-*n*-butane, and dodecafluoro-*n*-pentanes. For all these compounds temperatures of 1,000 °C or above were required for decomposition. With hexafluoropropane the activation energy for decomposition was 84 kcal/mole. A low value of 51 kcal/mole found for hexafluoroethane was suspected to be the result of heterogeneous reaction with the platinum filament used for pyrolysis. The compounds studied exhibited the following order of thermal stability:

$${\mathrm{CF}}_{4}>C_{2}F_{6}>\mathrm{cyclo}\;C_{5}F_{10}>C_{3}F_{8}>nC_{4}F_{10}>nC_{5}F_{12}$$

All of these compounds are much more thermally stable than the telomers of hexafluoropropylene, which decomposed readily at 350 °C.

Recent work [30, 31] on the thermal decomposition of fluoroaromatic compounds has resulted in establishing the following approximate order of stability for certain relative hydro and fluoroaromaticscontaining substances:

$$\begin{array}{c} (C_{6}F_{5}{)}_{2}>(C_{6}H_{5}{)}_{2}>C_{6}F_{5}-C_{6}H_{5}>(C_{6}H_{5}{)}_{2}O>\mathrm{Si}(C_{6}H_{5}{)}_{4}\\ >C_{6}F_{4}O_{2}C_{6}F_{4}>\mathrm{Si}(C_{6}F_{5}{)}_{4}>C_{6}F_{5}-O-C_{6}H_{5}\\ >P(C_{6}F_{5}{)}_{3}>P(C_{6}H_{5}{)}_{3}>(C_{6}F_{5}{)}_{3}\mathrm{PO}. \end{array}$$

It is seen that the effect of fluorination depends on the other elements present and on the structure of the molecules. Thus, where the breakdown may be due to the reaction or dissociation of a hydrogen atom, as in diphenyl, fluorine substitution improves the thermal stability. With the tetraphenyl silane the substitution of fluorine decreases the stability, probably by weakening the C—Si bonds. On the other hand, in triphenyl phosphine the effect of fluorine is to make the material more stable, whereas in the oxide form it becomes less stable. The P—C bond is probably stronger in the trispentafluorophenyl phosphine but weaker in the trispentafluorophenyl phosphine oxide compared to triphenyl phosphine. It may be recalled that perfluorotrialkyl amines are not basic and are quite inert and stable materials, as are the perfluorodialkyl ethers. Although detailed thermal decomposition studies have apparently not yet been carried out on these compounds, they are known to be quite stable. In general, from the listing of the compounds shown above and from other fragmentary data, it seems likely that perfluoroaryl ethers are likely to be quite stable, i.e., better than the hydrocarbon analogues. It is interesting to note that, as expected, mixed fluoro-hydro materials are less stable than either the straight fluoro or hydro substances.

### Photoinduced Decomposition of Polytrifluorochloroethylene

**Figure 15:**
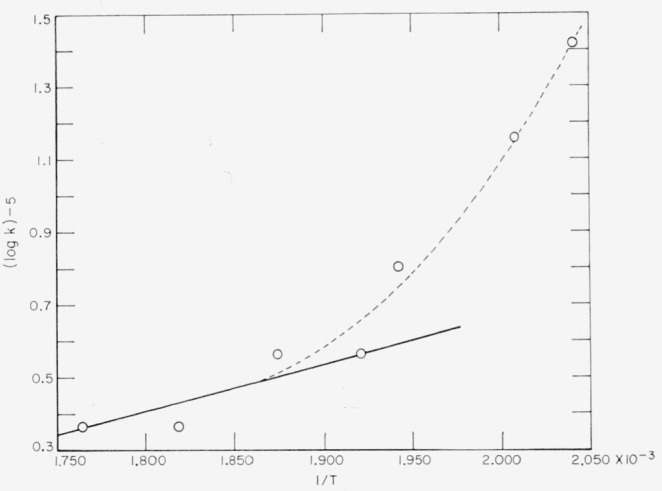
Arrhenius plot of post-irradiation decay of depolymerization rates ○, experimental points

A preliminary exploration of the photoinduced decomposition of polytrifluorochlorethylene was mentioned earlier, and plots of the reciprocal of rate of depolymerization, V, during the post-irradiation period were shown in figure 12. The linear character of the curves indicates that termination of the radicals is bimolecular. An Arrhenius plot of the slope of the lines in figure 12 yields a curious result (fig. 15). It is suggested that a diffusion effect on the depropagation step may be complicating the mechansism at lower temperatures. A similar result has been seen [32] on the activation energy for the photodecomposition of polymethyl methacrylate. The Arrhenius plot for the photoinduced depolymerization gave a fairly straight line with *E_ph_*=13 kcal/mole.

The activation energy for the thermal decomposition, *E_th_*, is 50 kcal/mole (see table 7). Assuming that transfer reactions are unimportant, *E_th_* is given by the relationship:

$$E_{th}=\frac{E_{1}}{2}+E_{2}-\frac{E_{4}}{2}$$

where *E*_1_ *E*_2_, and *E*_4_ are the activation energies, respectively, for the three elementary processes of the thermal depolymerization mechanism, — initiation, depropagation, and termination. In photoinduced depolymerization, initiation becomes indedependent of temperature, and hence *E_ph_=E*_2_ − (*E*_4_*/*2). The activation energy from the postirradiation decay, *E*_post_, i.e., the value derived from the slope in figure 15, is also related to *E*_2_ and *E*_4_*; E*_post_*=E*_4_−*E*_2_.

The plot in figure 15 suggests that *E*_post_ at high temperatures, near those where thermal decomposition occurs, is near zero. However, the curvature suggests that at the lower temperatures *E*_2_ becomes greater, since it is unreasonable to believe that *E*_4_ could become smaller. This suggests that monomer is finding it much more difficult to diffuse away from the radicals. The observed effect correlates nicely with the usual molding temperature or nostrength temperature.

The arbitrary straight full line in figure 15 yields *E*_post_= − 3 kcal, which we shall assume in making subsequent estimates. The dashed line shows the trend as temperature decreases. We can now estimate the values of *E*_1_ = 74, *E*_2_=23, and *E*_4_=20 kcal/mole for the elementary processes which comprise the mechanism of thermal decomposition for polytrifluorochloroethylene. It is believed likely that initiation occurs at random for both the thermal and the photoinduced decomposition of polytrifluorochloroethylene as the result of a dissociation of a chlorine atom from the chain. The dissociation energy of the polymer into radicals should be given by *D*(*R*−*R*)=*E*_1_−*E*_4_=54 kcal/mole [28]. The value obtained is reasonable for a secondary C−Cl bond dissociation.

Much further work is needed on the thermal decomposition of fluoro compounds and polymers. The present investigation has, however, pointed out the general features of their mechanisms of decomposition and certain differences and similarities between the fluorocarbons and the hydrocarbon polymers.

## Figures and Tables

**Table 1:** Pyrolysis of perfluoroamidine polymers a

Polymer	Temperature	Weight of sample	Total volatilized products	Light volatiles b

	*°C*	*mg*	%	%
A1	$\{\;\begin{array}{l} 430\\ 470\\ 503 \end{array}$	20.4	3.5	70.4
		22.3	6.5	68.3
		18.5	13.8	66.7
A2	$\{\;\begin{array}{r} 415\\ 455\\ 489\\ 505 \end{array}$	32.6	3.5	41.1
		27.4	6.0	34.5
		30.8	13.0	40.0
		20.9	21.1	46.0
A3	$\{\;\begin{array}{r} 470\\ 510 \end{array}$	26.3	31.0	12.5
		20.9	62.7	15.9

a Thirty-minute periods of pyrolysis.

b Based on amount of total volatiles.

**Table 2:** Representative analysis of light volatiles from pyrolysis of perfluoroamidine polymers

Components	A1	A2	A3

	*Mole* %	*Mole* %	*Mole* %
C_5_F_10_	……………	……………	0.3
C_4_F_8_	……………	……………	.3
C_3_F_8_	……………	0.9	1.5
C_3_F_6_	1.0	2.6	3.7
C_2_F_6_	……………	14.0	3.6
C_2_F_4_	75.7	51.6	34.7
CF_4_	6.3	17.3	17.5
SiF_4_	……………	0.7	15.0
CO_2_	13.7	12.9	23.4
C_4_H_8_	0.7	……………	……………
C_3_H_6_	.9	……………	……………
C_2_H_4_	1.7	……………	……………

**Table 3:** Pyrolysis of fluorinated polymers^a^

Polymer	Temperature	Wt. Of sample	Total volatilized products	Light volatiles b

	*°C*	*mg*	%	%
Polyhexafluoropentyleneadipate	$\{\;\begin{array}{r} 350\\ 415 \end{array}$	28.4	39.5	3.9
		28.4	98.9	2.6
FEP 100 X	$\{\;\begin{array}{r} 455\\ 478\\ 505\\ 545 \end{array}$	38.5	10.8	83.9
		30.8	21.8	83.1
		21.5	39.4	79.2
		18.4	92.4	75.0
VFHP-A	$\{\;\begin{array}{r} 385\\ 405\\ 500 \end{array}$	16.4	12.5	40.0
		30.9	57.1	34.7
		19.8	94.9	30.0
F 3700	$\{\;\begin{array}{r} 355\\ 380\\ 405 \end{array}$	22.5	4.5	32.0
		17.6	24.2	25.5
		21.5	85.4	23.1
F 5500	$\{\;\begin{array}{r} 375\\ 405\\ 435 \end{array}$	24.6	10.8	17.4
		21.1	56.6	15.7
		19.3	99.2	10.6

a Thirty-minute periods of pyrolysis.

b Based on amount of total volatiles.

**Table 4:** Representative analysis of light volatiles from the pyrolysis of various fluoropolymers in mole percent from mass spectrometer

Components	Hexafluoropentyleneadipate polymer^a^	FEP 100–X	VFHP–A	F elastomers

	400 °C	478 °C	400 °C	400 °C
C_4_F_8_	……………	2.0	……………	……………
C_3_F_8_	……………	0.4	……………	……………
C_3_F_6_	……………	54.8	……………	……………
C_2_F_6_	……………	0.5	……………	……………
C_2_F_4_	……………	35.6	……………	……………
SiF_4_	……………	1.3	80.0	80.3
C_6_H_12_	2.8	……………	……………	……………
C_4_H_8_	12.7	……………	……………	……………
C_2_H_4_	7.8	……………	……………	……………
CH_4_	3.3	……………	……………	……………
CO_2_	73.4	5.4	14.7	7.8
HCl	……………	……………	……………	11.9
SO_2_	……………	……………	5.3	……………
Totals	100.0	100.0	100.0	100.0

a For this polymer, about 30 mole percent of the light volatiles were not identifiable.

**Table 5:** Mole ratio of the methyl to argon ions in the mass spectra of the pyrolysis products of telomer PPF9A

Temperature	Time of pyrolysis	${\mathrm{CF}}_{3}^{+}/A$

°C	*hr*	
200	$\{\;\begin{array}{r} 4\\ 8\\ 24 \end{array}$	0.0012
		.0029
		.0115
225	$\{\;\begin{array}{r} 2\\ 4\\ 24 \end{array}$	.000^a^
		.0054
		.0630^a^
250	$\{\;\begin{array}{r} 2\\ 24 \end{array}$	.0948
		.2830
275	$\{\;\begin{array}{r} 2\\ 4\\ 24 \end{array}$	.3700
		.5898
		.4430
300	$\{\;\begin{array}{r} 2\\ 4\\ 8\\ 24 \end{array}$	.28
		.35
		.36
		.52
350	$\{\;\begin{array}{r} 2\\ 4\\ 8\\ 24 \end{array}$	1.1
		1.1
		1.0
		0.9
350	$\{\;\begin{array}{r} 2\\ 4\\ 8\\ 24 \end{array}$	1.2^a^
		1.3^a^
		1.3^a^
		1.3^a^
400	$\{\;\begin{array}{r} 2\\ 4\\ 8\\ 24 \end{array}$	1.0
		0.9
		.9
		1.0

a Nickel ampule.

**Table 6:** Mole ratio of hexafluoropropylene produced from pyrolysis at 225 °C to internal reference gas, argon

Telomer	Time of pyrolysis	Hexafluoropropylene/argon

	*hr*	
PPF6.5A	$\{\;\begin{array}{r} 2\\ 2\\ 24 \end{array}$	0.001
		.005
		.019
PPC16A	$\{\;\begin{array}{r} 2\\ 4\\ 24 \end{array}$	.003
		.008
		.029
PPC2A	$\{\;\begin{array}{r} 2\\ 4\\ 24 \end{array}$	…………..
		.014
		.069
BVFC5A	$\{\;\begin{array}{r} 2\\ 4\\ 24 \end{array}$	…………..
		…………..
		.001

**Table 7:** Thermal decomposition of fluorocarbon polymers

Polymer unit	Monomer and other major products	Total light volatiles	Rate, 350 °C.	E	A (est)

	*wt %*	*wt %*	*min.* %	*kcal/mole*	*sec*^−1^
CF_2_CF_2_ [3]	97 C_2_F_4_	100	2×10^−6^	81	10^19^
CF_2_CF [10]	73 CF_2_CF	74	4.8	64	10^19^
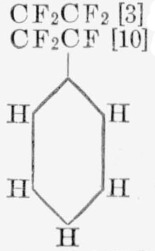	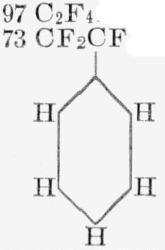				
CF_2_CFCl [10]	26 CF_2_CFCl	28	0.2	50	10^12^
CF_2_CFH [3]	7 HF	13	.02	53	10^12^
CF_2_CH_2_ [25]	13 HF	25	.02	48	10^10^
CH_2_CHF [3]	7.2 HF	18	.1	…………..	…………..

**Table 8:** Thermal decomposition of fluorocarbon copolymers

Polymer	Monomer and other major functions	Total light volatiles	Rate 350 °C	*E*	*A* (est)

	*wt* %	*wt* %	%/*min*	*kcal/mole*	*see*^−1^
FEP 100 X	52 C_2_F_4_, 22 C_3_F_6_	81	2×10^−4^	55	10^12^
Polymer A1	57 C_2_F_4_, 5 CO_2_, 4 CF_4_	68	6×10^−4^	39	10^6^
Polymer A2	21 C_2_F_4_, 8 C_2_F_6_, 6 CF_4_, 2 CO_2_	40	6×10^−4^	43	10^8^
Polymer A3	5 C_2_F_4_, 2 CF_4_, 2 CO_2_	13	8×10^−3^	31	10^5^
Polyhexafluoropentyleneadipate	1 C_4_H_8_, 2 CO_2_	3	2.0	32	10^8^
VFHP-A	24 HF	28	0.04	57	10^13^
F 3700	16 HF, 1.0 HCl	18	.06	61	10^15^
F 5500	10 HF, 1.2 HCl	13	.06	61	10^15^

## Footnotes

1 Figures in brackets indicate the literature references at the end of this paper.
